# Bottlenecks for High Coverage of Intermittent Preventive Treatment in Pregnancy: The Case of Adolescent Pregnancies in Rural Burkina Faso

**DOI:** 10.1371/journal.pone.0012013

**Published:** 2010-08-06

**Authors:** Koen Peeters Grietens, Sabine Gies, Sheick Oumar Coulibaly, Clotilde Ky, Judith Somda, Elizabeth Toomer, Joan Muela Ribera, Umberto D'Alessandro

**Affiliations:** 1 Prince Leopold Institute for Tropical Medicine, Antwerp, Belgium; 2 PASS International, Tessenderlo, Belgium; 3 UFR Sciences de la Santé, Université de Ouagadougou, Ouagadougou, Burkina Faso; 4 Laboratoire National de Santé Publique, Ouagadougou, Burkina Faso; Walter and Eliza Hall Institute of Medical Research, Australia

## Abstract

**Background:**

While IPTp-SP is currently being scaled up in sub-Saharan Africa (SSA), the coverage with the required ≥2 doses of SP remains considerably short of the Roll Back Malaria (RBM) goal of 80%, not to mention of the recently advocated universal coverage.

**Methods:**

The study triangulates quantitative data from a health center randomized community-based trial on IPTp-SP effectiveness and the additional benefit of a promotional campaign with qualitative data from focused ethnography.

**Findings:**

In rural Burkina Faso, despite the significantly higher risk of malaria infection among adolescent primigravidae (PG) (OR 2.44 95%CI 1.81–3.28, p<0.001), making them primary target beneficiaries of IPTp-SP, adolescents adhered to the required three or more ANC visits significantly less (PG: 46.6%; SG 43.7%) than adults (PG: 61.9%; SG 54.9%) and had lower SP uptake during the malaria transmission season, further showing the difficulty of reaching this age group. Adolescents' structural constraints (such as their social position and household labor requirements) and needs (such as anonymity in the health encounter) leave them highly vulnerable during their pregnancies and, especially, during the high malaria transmission season.

**Conclusion:**

Our study shows that adolescents need to be targeted specifically, prior to their first pregnancy and with measures adapted to their social context, addressing their structural constraints and needs and going beyond standard health promotion campaigns. Unless such specific measures are taken, adolescents' social vulnerability will present a serious bottleneck for the effectiveness of IPTi-SP.

## Introduction

In areas of high transmission, intermittent preventive treatment with sulfadoxine-pyrimethamine (IPTp-SP), is recommended for all pregnant women and is one of the cornerstones of malaria control together with insecticide treated bed nets (ITNs) and effective case management [1]–[5]. While IPTp-SP is being scaled up in sub-Saharan Africa (SSA) and despite high uptake of the first dose, the coverage with the required ≥2 doses of SP falls significantly short of the Roll Back Malaria (RBM) goal of 80%, not to mention of the recently advocated universal coverage [6]–[9]. There is, therefore, an urgent need to identify possible bottlenecks in order to allow this public health intervention to be efficiently translated into daily practice.

IPTp-SP delivery is closely linked to the access and utilisation of antenatal clinics (ANC), but little is known about the factors influencing the utilisation of antenatal health care, SP uptake and the interface between both factors [10]. Insufficient ANC attendance has been related to numerous factors such as distance, perceived inadequacy of services and high costs, and lack of privacy [11], [12]. Infrequent use of ANC and late attendance during pregnancy, the latter influencing the number of SP doses a woman can take, have also been related to education, low socio-economic status, high parity and unplanned or mistimed pregnancies [13]. At the health facility level, poor health worker performance, drug shortage, confusion about timing and spacing of SP doses, and correct assessment of the gestational age have further been identified as additional obstacles [14]–[17]. Among pregnant women, primigravidae, many of them still adolescents, have an increased susceptibility to malaria and are, therefore, one of the main beneficiary groups for IPTp-SP [18].

In this paper we describe age and parity specific patterns of SP uptake in the context of a targeted health promotion campaign that was conducted in rural Burkina Faso in order to stimulate ANC attendance and IPTp-SP adherence. Quantitative and qualitative methods are combined to explore and discuss socio-cultural aspects that set adolescents apart from other pregnant women and constitute bottlenecks for IPTp-SP coverage.

## Methods

### Study Site & Population

This study was conducted between 2003 and 2006, in Western Burkina Faso, in the rural health district of Boromo. In 2004, the estimated population of 204,117 people was distributed in 133 villages and 37 hamlets belonging to a variety of ethnic groups (*Bwaba*, *Dafing*, *Ko*, *Nounouma*, *Mossi*, *Peulh*, and others). Almost all practiced subsistence farming (sorghum, millet, maize, peanuts) and growing cotton for cash income. The climate is of Sudano-Sahelian type with a rainy season from May/June through October (mean rainfall 800 mm/year). Malaria is holoendemic with highly seasonal transmission during and after the rains. Formal health services are provided by a district hospital situated in the provincial capital Boromo and 27 peripheral health centers (HCs) situated in larger villages up to 85 km from Boromo.

### Study Design & Research Strategy

**Figure 1:**
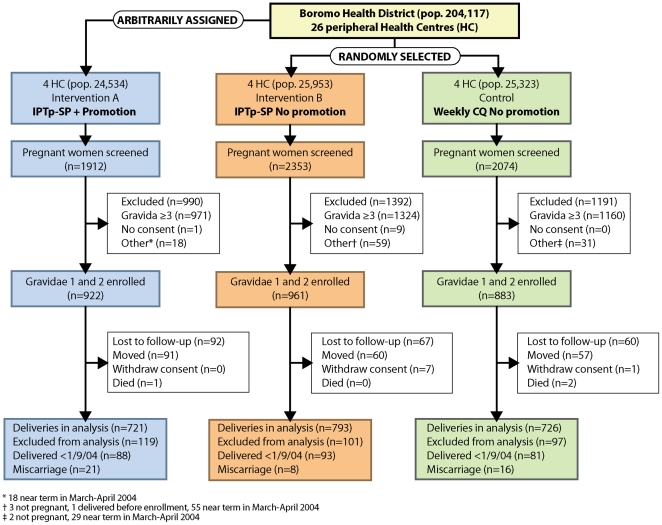
Trial participant flow diagram.

The health center randomized community-based trial on IPTp-SP effectiveness and the additional benefit of a promotional campaign has been described in detail elsewhere [19]. Briefly, twelve peripheral HCs were selected; in 8 of which IPTp-SP (2 doses, one in the second, and one in the third trimester of pregnancy) was introduced at ANC while the remaining HCs offered weekly chloroquine (CQ) according to the national policy at that time. Within the catchment areas of 4 HC introducing IPTp-SP, specially targeted promotional activities to enhance ANC attendance and SP uptake were conducted by community promoters [20] (Figure 1). These promoters were selected among local female leaders and trained to use pictorial material explaining all relevant aspects of ANC and IPTp-SP. They organized regular information sessions with groups of women in the intervention villages targeting all women of reproductive age and were supervised by a social scientist. During the study period, a new HC was opened in the study area, increasing the total number of study HCs to thirteen. Pregnant women were identified by women field assistants (WFA) through monthly rounds in the villages and encouraged to attend ANC if they were not yet booked. All consenting primigravidae (PG) and secundigravidae (SG) were enrolled and followed up until delivery.

Qualitative data were obtained during a targeted ethnography consisting of four months of field work in the study area between February and June 2006. Prior to the field research, a theoretical framework on IPTp, antenatal health care and the interface between both was elaborated, constructing two models for studying the social science aspects of malaria in pregnancy in order to provide initial categories guiding, though not determining, field work [10].

This article focuses on those aspects of IPTp-SP that set adolescents apart from other pregnant women. Problems of access to ANC and IPTp-SP that are common to all age groups, such as distance, real and perceived costs, the risk perception and perceived benefits of treatment and socio-economic status, are not presented here.

### Quantitative Data Collection

Women in their first or second pregnancy were identified at the community level by WFA and followed through pregnancy until delivery. Information on socio-demographic characteristics and on the current pregnancy was collected at enrolment and at follow-up visits (32 weeks of gestation and delivery) using structured questionnaires. Intake of SP tablets during ANC was supervised by health staff and recorded on study questionnaires. Thick blood films were prepared at ANC booking visits prior to the administration of antimalarial treatment. Gestational age at first ANC visit was estimated using the time between the first ANC and delivery, assuming delivery occurred after 40 weeks gestation. WFA visited participants likely to deliver at home weekly around the expected date of birth in order to record delivery data and birth weight as early as possible. Delivery data were equally collected at HC and at district hospital referrals.

### Qualitative Data Collection

Ethnographic field work was conducted in ten selected communities, representing all three intervention arms, using participant observation, interviews and group discussions.

#### Participant observation

Observation of people's behavior in their natural setting is a fundamental and often neglected part of qualitative research. For participant observation, clusters of adjacent villages were theoretically selected for more in-depth field work. Main selection criteria were the project's intervention and control branches (IPTp-SP plus promotion campaign | IPTp-SP without promotion campaign | weekly CQ) (for more detail see [20]); access to ANC and the presence of outreach programs; communities' perceived performance of the health center; reliability of contacts and informants; and, heterogeneity of the population according to ethnicity and religion. Short field stays (around 3–5 days) were repeatedly carried out in the selected clusters (Vy and Ouahabou; Serena and Zinakongo; Mou and Seyou; Toné and Laro Bidouané; and Bana and Wona). Reiterated and shorter visits and conversations were preferred as they establish better relations with informants and provide more reliable information than do longer stays where the researcher's presence risks becoming taxing or imposing. Additionally, all 13 HC in the study area were visited.

#### Interviewing

General and in-depth interviews were held in the selected locations. Interviews were recorded and transcribed after respondents' verbal consent. In those cases when the interviewer(s) considered that recording or taking notes in the presence of the respondent was not appropriate due to the sensitive nature of the subjects discussed, the required informality of the interview, the respondents' preferences or other limitations, the conversation was not recorded but the content written down immediately after the interview. 48 interviews were carried out with health staff and 35 with other community members and key informants (not including informal interviewing).

#### Group discussions

Both formal and informal group discussions were carried out in combination with the above-mentioned techniques. Group discussions were held with community promoters trained for the health promotion campaign (4GD; N = 12); pregnant women and extended family members that belong to the same *cour* (literally courtyard), forming a unit of social and economic cooperation (9GD; N = 32).

#### Structural characteristics of the data collection techniques

The degree of formality/informality and pre-defined structure of the mentioned research techniques was decided upon by the researchers in each specific context and based on the criteria of reliability of data. In general terms, research techniques were used in a less structured way in the beginning of the study and gradually defined while insights and preliminary knowledge was gathered. Reiterated informal conversations were held with the same respondents, especially adolescents, to increase trust and reduce bias - a possible problem since respondents generally tend to claim adherence to prescribed public health interventions, irrespective of their personal experience, opinions or actions, in order to avoid being seen as ‘negligent’ and/or ‘ignorant’ of basic biomedical health ordinances. A record of the most important informal conversations was kept until saturation.

#### Sampling

Sampling in the study localities was purposive. Informants were segmented according to relevant criteria such as gender, age, religion, ethnicity, locality, number of pregnancies, social acceptability of the pregnancy, access to ANC, timing of ANC-visits, etc. to allow for maximum variety and internal diversity, including critical cases. Guided observation, conversations, interviews and group discussions were held in all study locations and among a sufficient number of respondents to obtain a coherent picture of the social setting and the local social context. Participants in group discussions were selected by respondent driven sampling based on the criteria of confidentiality

### Data Analysis

#### Quantitative data

Data were double entered in an Access 2003 database and analyzed using Epi Info 2000 (version 3.2.2; Centers for Disease Control and Prevention, Atlanta) and STATA (Intercooled version 10; Stata Corp., College Station, TX) software packages. Differences in proportions were tested with the chi-square test and a p-value of <0.05 was considered statistically significant. Linear regression was used to compare means. Odds ratios (OR) with corresponding 95% confidence intervals (95%CI) were computed using logistic regression and adjusted for the cluster randomized design of the survey. Women experiencing a miscarriage were not included in the analysis. Gestational age was computed only for women with singleton live-births and analyses involving SP-uptake are restricted to 1,514 women who delivered in IPTp-SP study arms.

#### Qualitative data

In accordance with the research strategy, data analysis was a flexible and iterative process: preliminary data from different techniques were collected and analyzed; further research was then conducted confirming or refuting temporary results until saturation was reached and data could be theoretically supported. Interviews were systemized and analyzed with N/Vivo Qualitative Analysis software (QSR International Pty Ltd. Cardigan UK).

#### Ethical clearance

The study was approved by the Burkina Faso Ministry of Health and the Ethical Committees of the Prince Leopold Institute of Tropical Medicine. Local health authorities and community leaders were informed about the study objectives and procedures for data collection. All study participants gave informed consent after explanation of the procedures in the local language. Focused ethnography followed the Code of Ethics of the American Anthropological Association [21], [22]. Oral consent was preferred, since the interviewees were not put at any risk of being harmed in their safety or psychological well-being and because the act of signing one's name when providing data can be considered a potential reason for mistrust.

## Results

### Baseline characteristics of participants in the intervention study

Within the community-based trial, 2,240 pregnant women were followed up until delivery; 1,235 (55.1%) of them were PG and the remaining 1,005 SG, equally distributed across the three study arms (Table 1). More than half (55%) of the women were adolescents (≤19 years) with about one third of PG younger than 18 (31.8%). Despite the young age, the majority (95.0%) were married. Only 474 (21.2%) women had received any formal education. Affiliation to ethnic and religious groups reflected the cultural variety in the study area. About half of the women lived in villages with a HC, a quarter in villages with a HC within 5 km and a quarter with a HC more than 5 km away (Table 1).

**Table 1:** Baseline characteristics of primi (PG)- and secundigravidae (SG) followed until delivery in Boromo Health District, Burkina Faso, 2004–2006.

		PG (n = 1,235)		SG (n = 1,005)	
Characteristic	Subcategory	N	%	N	%
Age (years)	≤17	393	31.8	30	3.0
	18–19	553	44.8	231	23.0
	≥20	289	23.4	743	73.9
	Missing	0		1	0.1
Matrimonial status	Married monogamous	852	69.0	693	69.0
	Married polygamous	288	23.3	296	29.5
	Single	95	7.7	16	1.6
Formal education	None	960	77.7	798	79.4
	Primary school (1–3 ys)	65	5.3	54	5.4
	Primary school (4–6 ys)	165	13.4	122	12.1
	Secondary and higher	40	3.2	28	2.8
	Missing	5	0.4	3	0.3
SES	Most poor	283	22.9	225	22.4
	Poor	344	27.9	253	25.2
	Less poor	300	24.3	258	25.7
	Least poor	295	23.9	259	25.8
	Missing	13	1.1	10	1.0
Ethnic group	Bwaba	460	37.3	351	34.9
	Dafing	210	17.0	192	19.1
	Ko	144	11.7	123	12.2
	Nounouma	149	12.1	100	10.0
	Mossi	142	11.5	128	12.7
	Peulh	70	5.7	55	5.5
	Other	60	4.9	56	5.6
Religion	Moslem	589	47.7	484	48.2
	Christian	230	18.6	200	19.9
	Traditional	414	33.5	321	31.9
	Missing	2	0.2	0	0
Residence	Village with HC*	619	50.1	551	54.8
	Next HC at ≤5 km	260	21.1	206	20.5
	Next HC at >5 km	356	28.8	248	24.7
Season of delivery	Low transmission	621	50.3	513	51.0
	High transmission	606	49.1	489	48.7
	Missing	8	0.7	3	0.3
Intervention arm	IPTp-SP**+promotion	407	33.0	314	31.2
	IPTp-SP alone	442	35.8	351	34.9
	Weekly CQ	386	31.3	340	33.8

*Health Center.

**Intermittent preventive treatment in pregnancy with sulfadoxine-pyrimethamine.

### Malaria infection

**Figure 2:**
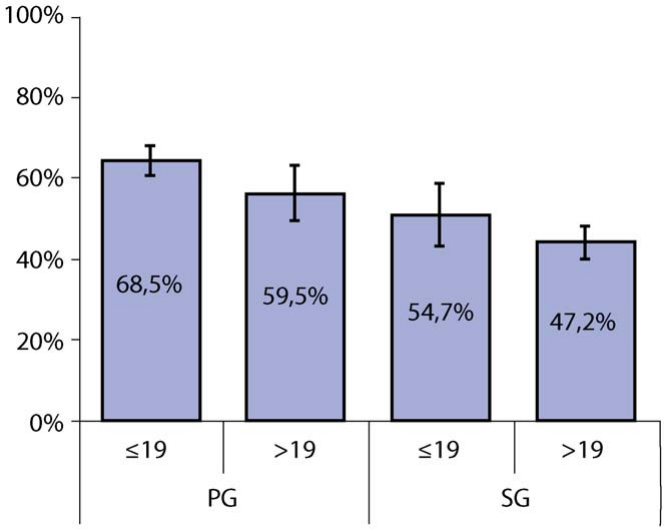
Positive malaria thick films at first antenatal visit in adolescent (≤19 years) and adult (>19 years) primigravidae (PG) and secundigravidae (SG).

Thick blood films were prepared during antenatal booking visits for 1,382 women (751 PG and 631 SG) prior to antimalarial treatment and the proportions of women with peripheral parasitaemia are presented in Figure 2. Overall, 809 (58.5%) women had malaria parasites, all *P.falciparum*, in the peripheral blood. The risk of being infected was lowest for adult SG followed by adolescent SG (OR 1.35 95%CI 0.93–1.96, p = 0.101). Adolescent PG had a more than two-fold increased risk of malaria infection (OR 2.44 95%CI 1.81–3.28, p<0.001) compared with adult SG. Among adults, PG had an equally increased risk when compared with SG (OR 1.64 95%CI 1.07–2.54, p = 0.028).

### Number of ANC Visits

All but 86 (3.8%) women attended antenatal clinics at least once during pregnancy, but only 51% completed the recommended three or more ANC visits. The total number of ANC visits was lower for adolescents (median [range] PG: 2 [0–6]; SG: 2 [0–5]) than adults (PG: 3 [0–5]; SG: 3 [0–6]) both in PG and in SG. The proportion of women having completed three or more ANC visits was significantly lower in adolescents (PG: 46.6%; SG 43.7%) than in adults (PG: 61.9%; SG 54.9%) (PG: OR 0.54; 95%CI 0.38–0.76, p = 0.002) (SG: OR 0.64; 95%CI 0.45–0.90, p = 0.015).

### Social factors related to the number of ANC-visits

Various social factors can account for fewer ANC visits among adolescents, most importantly (i) the social position of the adolescent mother; (ii) the social acknowledgement process of the pregnancy, especially in the case of PG; and, (iii) the cultural sense of shame or embarrassment, especially relevant when bringing the pregnancy into (iv) the public space of the health center.

#### Adolescents' social position

In the study area, settlements consist of patrilineally (kinship through the father's line) and patrilocally (in the place of residence of the husband) structured extended family clusters living in contingent or independent homes usually situated around the same *cour* or courtyard. Family members belonging to the same cluster function as a domestic group and can be referred to as belonging to this *cour* (*Zaka* in Moré and *Lou* in Dioula). Marriage can be born of individual initiative or arranged by the future spouses' families and exists with the option of further polygamous unions both in traditional religions and Islam. The *cour* and its members are of foremost importance for the recognition, acknowledgement and care of the pregnancy and the children born in the family cluster. Such patrilocal residence patterns imply that the woman, after marriage, will live with her husband's family and that the authority in her new household, especially when the couple is young, is in the hands of the parents-in-law, as household related authority is defined by gender (principally male>female) and age (older>younger). This limits adolescents' bargaining power and is especially relevant for intra- and inter-household labor substitution: when adult women of the same *cour* have work obligations in addition to other activities outside the *cour* (such as attending health promotion activities, antenatal care …), adolescents are required to tend to the additional domestic activities.

#### Social acknowledgement of the pregnancy

Directly related to the number of ANC-visits and possible delays, especially with first pregnancies, is the period during which the pregnancy remains hidden and is not socially acknowledged. This initial stage, between the first signs of pregnancy and its acknowledgement -‘*until the belly shows*’- has specific characteristics. First, early pregnancy is considered highly fragile and vulnerable and it is, therefore, considered dangerous/imprudent to reveal it at this early stage. Due to this initial vulnerability, strict laws of behavior apply, such as avoiding upsetting or insulting people, avoiding certain foods and places. These rules include not making the pregnancy public, especially outside the *cour*, which has a direct impact on early ANC attendance (Table 2.1).

**Table 2:** Quotes illustrating major findings.

**2.1. Social acknowledgement of the pregnancy and related vulnerability**
Q1. The pregnant woman protects herself by being wary and suspicious. [Interview mother (Siby)]
Q2. The habit here, [for the first pregnancy,] is that when you are asleep at night, one of the women [of the *cour*] will come and she will call you three times, four times and then you answer. Some time later, when the traditional customs are finished, they will dress you in a white traditional cloth [*pagne*] to protect the child. No one should tell you you're pregnant before this moment, otherwise you will lose the child. (…) After this you can go to the ANC. [Interview adolescent mother (Mou)]
Q3. When women are pregnant, we carry out rituals, especially for young women that just got married and are pregnant for the first time. When there are no problems with her pregnancy, this is after 3 to 4 months (…) They can go to the ANC before this but it is true that most will not, they will wait 3 to 4 months because they are wary of other people finding out. (…) She has to hide herself because until the belly has gotten big, other women should not know, and the men either, they shouldn't know. [Interview with Mossi Mother (Laro Bidouané)]
**2.2. Shame and embarrassment**
Q4. For your first pregnancy, you're always going to be ashamed. You're ashamed, it's something you've never seen, and suddenly you're belly starts growing and everyone will find out that you've been with a man and that everything will change. [Adolescent in GD (Siby)]
Q5. Women do come to the ANC but those who already have a small child and are pregnant again, they won't come. You talk and talk but still they will not come! [Interview health promoter (Serena)]
Q6. When your child is still small and you're already pregnant again people will say that it's because the husband ‘likes’ his wife too much or that the wife doesn't want the husband to leave the house. [Adolescent in GD (Toné)]
**2.3. The health centre as public space**
Q7. There will be so much talk that she is ‘loose’ and that's why she's pregnant: that she isn't married yet and that she got pregnant while still living in the *cour* of her father: that she was too ‘eager’ … Many things like this will be said. Once you go to the health centre, everyone will talk. [Adolescent in GD (Toné)]
Q8. When you hide that you're pregnant even when you get ill, you don't want to go to the health centre. You have to hide yourself when you go there to buy the medication! [Interview adolescent Mossi (Laro Biduoané)]
Q9. When I go to the ANC, everyone will know that I'm pregnant because they have seen me at the maternity ward. When someone is pregnant, often even the other women of the same *cour* do not know! I will wait until they all know that I'm pregnant before going to the ANC. [Interview pregnant adolescent (Toné)]
Q10. Some women they get married but ‘go’ somewhere else, so when she is pregnant, she can not go to the ANC. Others are pregnant but they still live with their father, or she is pregnant but not from her husband, so she's ashamed. She can not go to the ANC. If they go to the ANC, the whole village will know. [Interview adolescent mother (Mou)]

After this initial phase, the pregnancy is socially acknowledged through the observation of certain rituals or simply by the unmistaken visibility of the belly. Common rituals ending this period of ambiguity and vulnerability are those that alert the ancestors of the woman's pregnancy and prompt their protection of the unborn child. These rituals include the ‘request for ancestors' protection’ (commonly seen in the symbolic shaving of the pregnant woman's head) and the ‘binding of the breasts’ (the wearing of a white dress “pagne” to ‘lower the breasts’ -e.g. *rungri* among the Mossi). The importance of such rituals derives from the fact that they lead to the protection of the pregnancy and consequently allow the woman to bring it into the open. From this point on, the woman is officially pregnant, putting an end to the initial highly vulnerable and ‘hidden’ stage of pregnancy. When no rituals are observed, the pregnancy is considered most vulnerable until the pregnant woman's belly is clearly visible and the pregnancy is subsequently socially acknowledged (Table 2.1). Furthermore, women attending the ANC earlier expect to be mocked by their female counterparts.

#### Shame

In line with the social acknowledgement of pregnancy, a culturally-determined sense of shame or embarrassment (*honte*) -understood as modesty and shyness related to a sense of propriety, stops adolescents from making their first pregnancies public until they can no longer be hidden. A key point in understanding this sense of shame or embarrassment is that pregnancy marks the passage from one stage of the adolescent's life to another; it is a key moment in the transition from childhood to adulthood accompanied by an intensification of culture transmission. When all attention is directed at the young woman when she is for the first time pregnant, she is expected to be modest and feel embarrassed. Additionally, socially less acceptable pregnancies, such as adolescents who are pregnant while still living in their father's *cour* or those with extra-marital and mistimed pregnancies (soon after a previous births) (Table 2.2), are considered shameful and are accompanied by related social problems, directly limiting women's access to ANC and consequently IPTp.

#### The health center as public space

Attending the ANC brings a woman's pregnancy into the public sphere where her actions and behavior are weighed and measured according to community expectations and existing social rules. This lack of privacy and confidentiality in the health encounter can make adolescents reluctant to attend ANC and publicize their pregnancies or can even directly stop adolescents from seeking antenatal care at an early stage of pregnancy. Additionally, health centers are often located in larger villages or towns near markets that are important meeting places. Consequently, women will only go to the health center if (i) they are ready to make their pregnancy public; or, (ii) if there is an acute health problem since going to the health center often amounts to an official disclosure of pregnancy (Table 2.3.).

### SP Uptake and the effect of the promotion campaign

**Figure 3:**
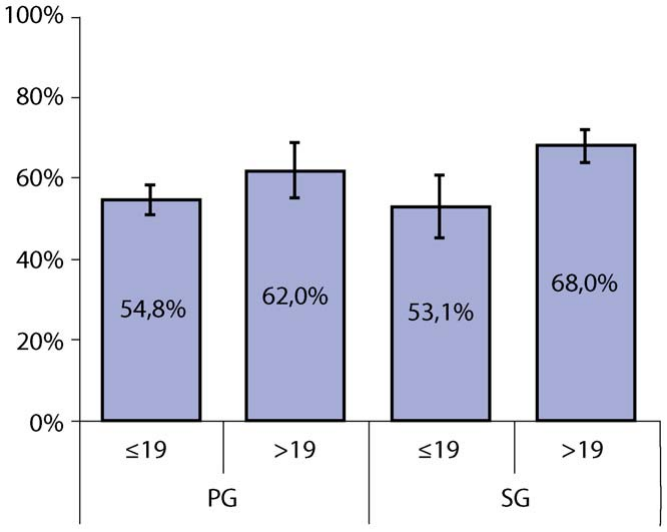
Proportion of women having received ≥2 doses of IPTp-SP during pregnancy by parity and age group. Error bars represent 95%confidence intervals. PG = primigravidae, SG = secundigravidae.

**Figure 4:**
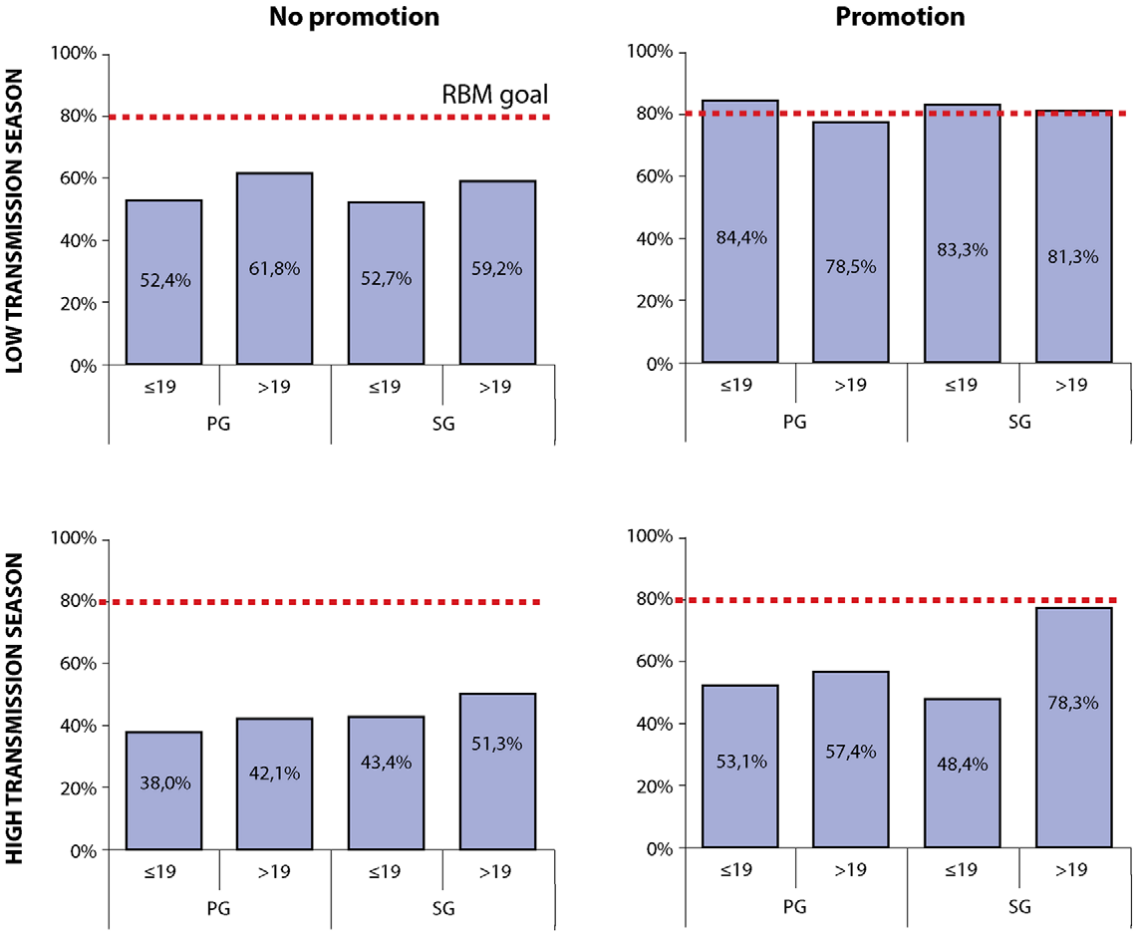
IPTp-SP uptake (≥2 doses) in adolescent (≤19) and adult (>19) primigravidae (PG) and secundigravidae (SG) in villages with and without promotion during the high and low transmission season. * difference compared with reference group (SG/>19) significant (p<0.05) in logistic regression analysis.

Overall, uptake of at least 2 doses of IPTp-SP was 60.0% and was significantly lower among adolescent PG (OR 0.57 95%CI 0.43–0.75, p = 0.001) and SG (OR 0.53 95%CI 0.32–0.87, p = 0.017) when compared to adult SG, but only slightly lower in adult PG (Figure 3). IPTp-SP uptake was significantly lower in women delivering during the high malaria transmission season when compared with the low transmission period (Figure 4 left side) (OR 0.61 95%CI 0.51–0.73, p<0.001 adjusted for age and parity). Therefore, the effect of the promotion campaign for improving IPTp-SP uptake is presented stratified by season (Figure 4 right side). During the low transmission season, SP uptake increased significantly in all age and parity groups (OR 3.63 95%CI 1.89–7.0, p = 0.001) with no major differences between adolescents and adults and almost all groups reaching the 80% coverage RBM goal. However, among women delivering during the high transmission season, adolescent PG had a lower SP uptake when compared to adult SG (OR 0.58 95%CI 0.39–0.86, p = 0.012). The effect of the promotion campaign during the high transmission season was of borderline significance (OR 2.11 95%CI 1.0–4.49, p = 0.051) and mainly due to increased SP uptake among adult SG. The uptake of SP was significantly lower in adolescent SG (OR 0.26 95%CI 0.09–0.78, p = 0.021) and both adult (OR 0.37 95%CI 0.18–0.75, p = 0.010) and adolescent PG (OR 0.31 95%CI 0.12–0.79, p = 0.019).

The reduced impact of the promotion campaign on SP uptake among adolescents, especially in the rainy season, can be related to the following factors. First, the promotion campaign did not directly target adolescents but, instead, addressed pregnant women in general. Therefore, in local communities, the campaign was considered of less direct interest to adolescents with pregnancies that are still not socially acknowledged or *hidden*. Second, internal authority and status regulations limit adolescent participation during the health promotion campaign when they are gathered in the same information sessions as adults. Third, especially during the work-intensive rainy season, when most of the income for the rest of the year is secured, intra and inter-household labor substitution requires additional domestic work from adolescents, negatively affecting adolescents' ANC attendance.

## Discussion

Despite the significantly higher risk of malaria infection among adolescent PG, making them primary target beneficiaries of IPTp-SP, adolescents completed the required three or more ANC visits significantly less than adults and had lower SP uptake during the malaria transmission season. The uptake of IPTp-SP was lowest in adolescent PG during the high malaria transmission season and was only marginally improved by health promotion activities. In contrast, good coverage was achieved in older SG despite otherwise similar socio-economic characteristics.

Moreover, while unmarried pregnant adolescents have been identified as a high risk group insufficiently reached by health interventions [5], our results shows that in rural Burkina Faso even married adolescents had impaired access to malaria prevention. Three kinds of factors were identified accounting for adolescents' vulnerability: (i) the adolescent's social position; (ii) the recognition process of the pregnancy - especially for PG; and, (iii) health system factors.

With regard to their social status, newly married adolescents have the most demanding social position in the *cour*. Household related authority is defined by gender and age, leading to limited bargaining power in the domestic negotiation process for adolescent females. In her new social position, the newly wed adolescent is subject to stricter social expectations, which include being a hard worker, respecting her in-laws, bearing children and accepting additional domestic tasks related to intra and inter-household labor distribution. Despite the fact that women have legitimate claims on common resources within the household, traditional norms and mechanisms of intra-household resource allocation have been shown to directly influence women's access to healthcare [25]. In this way, married female adolescents are less mobile and their mobility is restricted to locations that allow them to fulfill household and child-care responsibilities [26]. Paradoxically, despite IPTp-SP being a child-care responsibility, there is less willingness to negotiate the adolescent woman's obligations and resources in the household when preventive care is needed than in cases where curative care is required, leading to a more precarious situation for the pregnant adolescent, especially in the first stage of her pregnancy and as long as no apparent problem occurs.

In terms of the public acknowledgment of pregnancies, a pregnant woman does not publicly disclose her condition at an early stage due to a sense of shame or embarrassment and rules of propriety, the pregnancy's vulnerability, the fact that publicizing a pregnancy can expose a woman to (magico-religious) harm and because pregnant women are expected to follow a certain number of rites, prescriptions and interdictions before the pregnancy is made common knowledge. This initial ‘hidden’ phase of the pregnancy has been reported in other contexts, such as in Gabon and Cameroon (Peeters Grietens, unpublished data) and The Gambia, where young women hide their pregnancies for as long as possible for fear of attracting evil or misfortune to their unborn child [27]. Late booking or non-attendance at antenatal clinics by young pregnant women [12], [23], [24] and low uptake of malaria preventive measures [28]–[30]) have also been reported in other studies and can jeopardize the effectiveness of preventive programs for malaria in pregnancy.

In relation to health system factors, maternal health policies are based on general indicators for ANC coverage and assisted delivery, which do not include (age-) specific sub-groups. As such, health policies and health promotion do not specifically target adolescents, leaving their needs unaddressed [31], [32] despite the fact that, in social settings such as in rural Burkina Faso, as many as three quarters of PG are adolescents.

The public health services' limitations in addressing the specific difficulties faced in adolescent pregnancies were apparent in the lack of anonymity and privacy during the health encounter, which represents one of the main barriers to adolescents' access to care in the study setting. Being a public space, attending the health center often amounts to an official disclosure of pregnancy while this might not be desired. This lack of confidentiality for adolescents in the health encounter has also been shown, in other contexts, to make adolescents reluctant to identify themselves as being pregnant [5], therefore hindering antenatal care attendance. Targeting adolescents directly and prior to their first pregnancy while assuring anonymity in access to and during the health encounter can contribute to meeting the needs of early ANC booking while respecting the socio-cultural demand of keeping early pregnancies hidden. Other health system factors that relate directly to adolescents' work activities such as appropriate opening hours, reduced HC waiting times, and adapted outreach programs, have to be further evaluated in order to address more structural constraints of access to ANC.

Finally, the data on the impact of the health promotion campaign in relation to seasonality provide us with valuable supplementary insights into the effectiveness of IPTp-SP. While the health promotion campaign improved ANC attendance and SP uptake during the dry season, it failed to bring about behavioral change during the rainy season when malaria is highest but work is also most demanding. This shows that although SP uptake can be improved through a combination of information campaigns and community involvement in health promotion during the dry season, other structural factors limiting adolescents' access to IPTp-SP, such as labor requirements and poverty, remain despite IEC-campaigns. This also suggests that rituals and possible beliefs, which are independent of the season and that can cause initial delays among adolescents, are more easily overcome with health promotion than are the structural constraints of adolescents' social position and the limitations of health services for adolescent care. In other words, the importance of seasonality illustrates that IEC campaigns alone are not enough to foster an effective IPTp-SP program for the most vulnerable groups in the season that they most need it. Seasonality merits more research as little is currently known about its importance for preventive health [33], [34] (i.e. how do work requirements and seasonal mobility patterns influence adherence to IPTp as farmers often reside at fields for longer periods when work is intensive, decreasing accessibility to the health center; Peeters Grietens unpublished data).

The lack of priority given to adolescents and the absence of a framework for delivering health and health-related interventions to this high-risk group has been repeatedly highlighted [35]. In line with these findings, we conclude that a general framework for delivering IPTp-SP to adolescents is urgently needed, reiterating the need for adolescent specific health care.

## References

[1] Parise ME, Ayisi JG, Nahlen BL, Schultz LJ, Roberts JM (1998). Efficacy of sulfadoxine-pyrimethamine for prevention of placental malaria in an area of Kenya with a high prevalence of malaria and human immunodeficiency virus infection.. Am J Trop Med Hyg.

[2] Schultz LJ, Steketee RW, Chitsulo L, Macheso A, Nyasulu Y (1994). Malaria and childbearing women in Malawi: knowledge, attitudes and practices.. Trop Med Parasitol.

[3] Shulman CE, Dorman EK, Cutts F, Kawuondo K, Bulmer JN (1999). Intermittent sulphadoxine-pyrimethamine to prevent severe anaemia secondary to malaria in pregnancy: a randomised placebo-controlled trial.. Lancet.

[4] Verhoeff FH, Brabin BJ, Chimsuku L, Kazembe P, Russel WB (1998). An evaluation of the effects of intermittent sulfadoxine-pyrimethamine treatment in pregnancy on parasite clearance and risk of low birthweight in rural Malawi.. Ann Trop Med Parasitol.

[5] World Health Organization (2004). Adolescent Pregnancy.. http://whqlibdoc.who.int/publications/2004/9241591455_eng.pdf.

[6] WHO (2008). World Malaria Report 2008.. http://apps.who.int/malaria/wmr2008/malaria2008.pdf.

[7] RBM (2005). Monitoring and Evaluation Reference Group (MERG)..

[8] World Health Organization (2005). Global strategy plan 2005–2010. Geneva, World Health Organization.. http://www.rollbackmalaria.org/forumV/docs/gsp_en.pdf.

[9] RBM (2008). RBM Partnership. The global malaria action plan.

[10] Ribera JM, Hausmann-Muela S, D'Alessandro U, Grietens KP (2007). Malaria in Pregnancy: What Can the Social Sciences Contribute?. PLoS Med.

[11] Ndyomugyenyi R, Neema S, Magnussen O (1998). The use of formal and informal services for antenatal care and malaria treatment in rural Uganda.. Health Policy and Planning.

[12] Okonofua FE, Feyisetan BJ, Davies-Adetugbo A, Sanusi YO (1992). Influence of socio-economic factors on the treatment and prevention of malaria in pregnant and non-pregnant adolescent girls in Nigeria.. J Trop Med and Hyg.

[13] Magadi MA, Madise NJ, Rodrigues RN (2000). Frequency and timing of antenatal care in Kenya: explaining the variations between women of different communities.. Soc Sci and Med.

[14] Crawley J, Hill J, Yartey J, Robalo M, Serufilira M (2007). From evidence to action? Challenges to policy change and programme delivery for malaria in pregnancy.. Lancet Infect Dis.

[15] Hill J, Kazembe P (2006). Reaching the Abuja target for intermittent preventive treatment of malaria in pregnancy in African women: a review of progress and operational challenges.. Trop Med Int Health.

[16] Newman RD, Moran AC, Kayentao K, Benga-De E, Yameogo M (2006). Prevention of malaria during pregnancy in West Africa: policy change and the power of subregional action.. Trop Med Int Health.

[17] Van Eijk AM, Ayisi JG, Kuile FO, ter Slutsker L, Otieno JA (2004). Implementation of intermittent preventive treatment with sulphadoxine-pyrimethamine for control of malaria in pregnancy in Kisumu, western Kenya.. Trop Med Int Health.

[18] Lalloo DG, Olukoya P, Olliaro P (2006). Malaria in adolescence: burden of disease, consequences, and opportunities for intervention.. Lancet Infect Dis.

[19] Gies S, Coulibaly SO, Ouattara FT, Ky C, Brabin BJ (2008). A community effectiveness trial of strategies promoting intermittent preventive treatment with sulphadoxine-pyrimethamine in pregnant women in rural Burkina Faso.. Malar J.

[20] Gies S, Coulibaly SO, Ouattara FT, Ky C, Brabin BJ (2009). Community-based promotional campaign to improve uptake of intermittent preventive antimalarial treatment in pregnancy in Burkina Faso.. Am J Trop Med Hyg.

[21] American Anthropological Association: Code of Ethics of the American Anthropological Association.. http://www.aaanet.org/committees/ethics/ethcode.htm.

[22] American Anthropological Association: American Anthropological Association Statement on Ethnography and Institutional Review Boards.. http://www.aaanet.org/stmts/irb.htm.

[23] Ojengbede OA, Otolorin EO, Fabanwo AO (1987). Pregnancy performance of Nigerian women aged 16 years and below, as seen in Ibadan, Nigeria.. Afr J Med Sci.

[24] De la Cruz AC (1996). Experience with teenage pregnancy at Eulogio Rodriguez, Sr. Memorial hospital.. J Philippine Med Association.

[25] Nikiéma B, Haddad S, Potvin L (2008). Women Bargaining to Seek Healthcare: Norms, Domestic Practices, and Implications in Rural Burkina Faso.. World Development.

[26] Brady M, Saloucou L (2007). Addressing the needs of married adolescent girls in Burkina Faso.. Transitions to Adulthood,.

[27] Stokes E, Dumbaya I, Owens S, Brabin L (2008). The right to remain silent: a qualitative study of the medical and social ramifications of pregnancy disclosure for Gambian women.. BJOG.

[28] Marchant TJ, Armstrong Schellenberg J, Edgar T, Nathan R, Abdulla S (2002). Socially-marketed insecticide-treated bednets improve malaria and anaemia in pregnancy in southern Tanzania.. Trop Med & Int Health.

[29] Nganda RY, Drakeley C, Reyburn H, Marchant T (2004). Knowledge of malaria influences the use of insecticide treated nets but not intermittent presumptive treatment by pregnant women in Tanzania.. Malar J.

[30] Van Eijk AM, Blokland IE, Slutsker L, Odhiambo F, Ayisi JG (2005). Use of intermittent preventive treatment for malaria in pregnancy in a rural area of western Kenya with high coverage of insecticide-treated bed nets.. Trop Med Int Health.

[31] Brabin L, Stokes E, Dumbaya I, Owens S (2009). Rural Gambian women's reliance on health workers to deliver sulphadoxine – pyrimethamine as recommended intermittent preventive treatment for malaria in pregnancy.. Malar J.

[32] Brabin L, Verhoeff FH, Kazembe P, Brabin BJ, Chimsuku L (1998). Improving antenatal care for pregnant adolescents in southern Malawi.. Acta Obstet Gynecol Scand.

[33] Hounton SH, Sombie I, Townend J, Ouedraogo T, Meda N (2008). The tip of the iceberg: evidence of seasonality in institutional maternal mortality and implications for health resources management in Burkina Faso.. Scand J Public Health.

[34] Sauerborn R, Nougtara A, Hien M, Diesfeld HJ (1996). Seasonal variations of household costs of illness in Burkina Faso.. Soc Sci Med.

[35] Brabin L, Brabin BJ (2005). HIV, malaria and beyond: reducing the disease burden of female adolescents.. Malar J.

